# Implications of Foot Ulceration in Hemodialysis Patients: A 5-Year Observational Study

**DOI:** 10.1155/2014/945075

**Published:** 2014-03-03

**Authors:** Hassan Al-Thani, Ayman El-Menyar, Valsa Koshy, Ahmed Hussein, Ahmed Sharaf, Mohammad Asim, Ahmed Sadek

**Affiliations:** ^1^Vascular Surgery, Department of Surgery, Hamad General Hospital, HMC, P.O. Box 3050, Doha, Qatar; ^2^Clinical Research, Trauma Surgery, Hamad General Hospital, P.O. Box 3050, Doha, Qatar; ^3^Clinical Medicine, Weill Cornell Medical College, P.O. Box 24144, Doha, Qatar; ^4^Cardiology Section, Internal Medicine, Ahmed Maher Teaching Hospital, Cairo, Egypt

## Abstract

Foot ulceration (FU) remains a serious concern for patients worldwide. We analyzed the incidence, risk factors, and outcome of FU in hemodialysis (HD) patients. A retrospective cohort study was conducted for 252 HD patients who were followed up for 5 years. Patients were categorized according to whether they developed FU or not. The FU group (17%) was older and had significantly higher incidence of nephropathy, retinopathy, peripheral (PAD), coronary artery disease (CAD), and diabetes mellitus (DM) as compared to no-FU group. FU group had higher frequency of major amputation (*P* = 0.001) and HD vascular access (*P* = 0.01). Patients with combined DM and PAD had a 10-fold increased risk of FU in comparison to those who had DM alone. Presence of PAD was the main independent predictor for development of FU in HD with an adjusted odd ratio (aOR) of 16.0 (95% CI: 4.41–62.18; *P* = 0.001). After adjusting for age, sex, and CAD, predictors for mortality were PAD (aOR 4.3), FU (aOR 3.6), and DM (aOR 2.6). FU is common in HD patients regardless of DM. However, the presence of PAD is significantly associated with more FU and mortality in HD. HD patients need intensive foot care and warrant progressive modification of vascular risk factors.

## 1. Introduction

It has been well established that patients with end-stage renal disease (ESRD) undergoing hemodialysis (HD) had a higher association of peripheral arterial disease (PAD), lower extremity amputation (LEA), and foot ulcerations (FU) [1–3]. Such an association could be attributed to a common atherosclerotic cause. Moreover, diabetic patients with ESRD had a 10-fold increased risk of LEA in comparison to diabetes patients without renal insufficiency [1]. Several studies have reported increased rates of LEA among ESRD patients, irrespective of the concomitant presence of diabetes mellitus (DM) [4–6].

The mechanism of FU development is multifactorial which depends upon various physiological, mechanical, and treatment factors. Hill et al. [7] reported significantly higher incidence of foot complications in patients with concomitant ESRD and DM in comparison to those who had only DM suggesting a possible relationship between ESRD and FU. In addition, LEA and FU are well established complication of diabetic nephropathy [8, 9]. Margolis et al. [10] observed a strong correlation between progressions of chronic kidney disease (CKD) with the development of diabetic FU. Game et al. [11] also demonstrated a close association of FU and amputations in diabetic patients started to undergo dialysis. Brownrigg et al. [12] performed a meta-analysis to look for the relationship of diabetic FU with cardiovascular disease (CVD) and all-cause mortality. The authors found an increased association of CVD and mortality in diabetic patients with FU in comparison to those without FU. Interestingly, a recent study advocated a higher incidence of risk factors (PAD and peripheral neuropathy) for FU among HD patients [13]. In Qatar, an earlier report from our center identified a high incidence of PAD in HD patients [14]. However, there is a lack of information that describes the risk factors and prognostic implications of FU among HD patients in the Arab Middle East. We aim to evaluate this association and its impact on the outcomes over a 5-year period.

## 2. Methods

We conducted a retrospective analysis of all hemodialysis patients enrolled at the HD unit at Hamad General Hospital (HGH), Qatar, over five years (2007–2012) duration. The study recruited 252 consecutive patients with ESRD who need regular HD. Patients surviving for at least 3 months on the initial HD were included in the study. The study excluded all patients that were on peritoneal dialysis or had incomplete data. HD patients were categorized into two groups according to whether they had foot ulcer (FU) or not (no-FU) based on physical examination. The attending physician in the HD unit collected the data regarding the demographics characteristics, clinical evaluation, medical history, and comorbidities. During the follow-up period over 5 years, HD-related procedures such as vascular access (arteriovenous fistula, arteriovenous grafting, and tunneled catheter), renal transplantation, and vascular complication were also reported. The need for ≥3 vascular accesses (due to mechanical obstruction, poor flow, or infection) had been analyzed. We speculated that HD patients with FU had poor outcomes compared to no-FU patients. The study end-points (major amputation, vascular complications, and mortality) were also subanalyzed according to the presence or absence of DM and PAD.

FU was defined according to the clinical findings as a full thickness skin break below the level of malleoli. Further microbiology and radiological assessment were done as well for ulcers. Patients were considered to have PAD if they had one of the following criteria: ABI < 0.9, history of intermittent claudication, vascular bypass or endovascular intervention for occlusive vascular disease, or amputation due to occlusive vascular disease [14]. Major amputations were defined as amputations proximal to the ankle joint, and minor amputations were defined as those through or distal to the ankle joint [15]. Diabetic nephropathy was diagnosed during patient follow-up in the nephrology out-patient clinic according to the patient chart. It was defined as presence of macroalbuminuria that is a urinary albumin excretion of more than 300 mg in a 24-hour collection or macroalbuminuria and abnormal renal function as represented by an abnormality in serum creatinine, calculated creatinine clearance, or glomerular filtration rate [16].

Data were presented as proportions or mean and standard deviation, as appropriate. Analyses were conducted using the Student's *t*-test for continuous variables and Pearson chi-square (*χ* ^2^) test for categorical variables. Multivariate logistic regression analysis was performed for predictors of development of FU and mortality. Adjusted Odds Ratios (OR), 95% CI, and *P* values were reported for significant predictors. A significant difference was considered when the two-tailed *P* value was <0.05. Data analysis was carried out using the Statistical Package for Social Sciences version 18 (SPSS Inc., Chicago, Illinois). The study has been approved by the Medical Research Center, Hamad Medical Corporation, Qatar (IRB #12007/12).

## 3. Results

**Figure 1:**
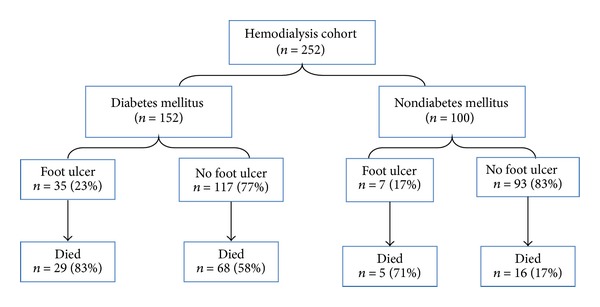
Study flow chart.

A cohort of 252 HD patients was included in the study, of which 42 had FU (17%) and 210 were without FU. Demographic, clinical characteristics, and risk factors for HD patients with and without FU were shown in Table 1 and Figure 1. Patients with FU were 6 years older and had higher incidence of retinopathy (67% versus 40.5%; *P* = 0.002), polyvascular disease (26.2% versus 6.7%; *P* = 0.001), angina (24% versus 11%; *P* = 0.02), PAD (71% versus 32%; *P* = 0.001), and nephropathy (79% versus 42%; *P* = 0.001) compared to no-FU patients. Also, a high percent of patients needed tunneled catheters (88% versus 82%; *P* = 0.03) and aspirin (62% versus 45%; *P* = 0.04) in the FU group, whereas, history of renal transplant (14% versus 2.4%; *P* = 0.03) was observed more in no-FU group. Patients in FU group also had higher percentage of DM (83.3% versus 56%; *P* = 0.001), major amputation (26% versus 1%; *P* = 0.001), need for ≥3 vascular accesses (69% versus 47%; *P* = 0.01), and coronary artery disease (45% versus 28%; *P* = 0.02) than no-FU group. The two groups were comparable regarding the mean baseline laboratory investigations (Table 2). Similarly, the percentage of dyslipidemia, hypertension, smoking, and cerebral vascular accident (CVA) was comparable between the two groups (Table 1). The types of HD vascular accesses such as arteriovenous fistulas, graft, or catheter were comparable in the 2 groups.

### 3.1. Clinical Outcomes at Three-Year Period

Overall mortality rate within 3 years was 24.2% among the study cohort and was comparable between the two groups. Development of PAD (71% versus 32%; *P* = 0.001), new foot ulcers (47.6% versus 2.4%; *P* = 0.001), and need for major amputation (16.7% versus 1%; *P* = 0.001) were significantly higher among FU group as compared to no-FU group.

### 3.2. Clinical Outcomes at Five-Year Period

In the following two years, significantly more number of patients in FU group developed new PAD (22% versus 1.4%; *P* = 0.001), underwent major amputation (22% versus 0%; *P* = 0.001), and died (70.4% versus 17.5%; *P* = 0.001) in comparison to no-FU group (Table 1). The frequency of renal transplant and angina was comparable among both the groups, while cerebral vascular accident (3.5%) was only observed in no-FU group.

**Figure 2:**
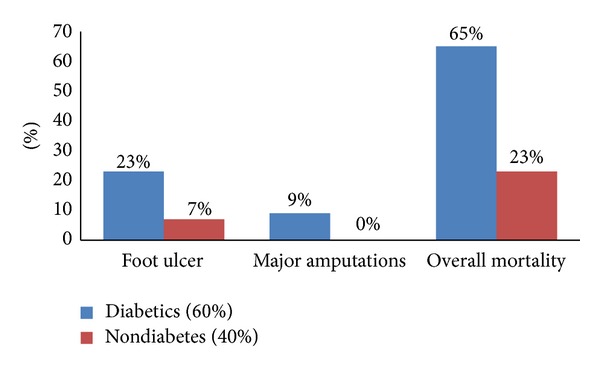
Outcomes in HD patients based on the presence of DM (*P* = 0.001 for all).

Figure 2 shows that DM patients had significantly higher incidence of FU (23% versus 7%; *P* = 0.001), need for major amputation (8.6% versus 0%; *P* = 0.003), and mortality (65% versus 23%; *P* = 0.001) than non-DM patients.

**Figure 3:**
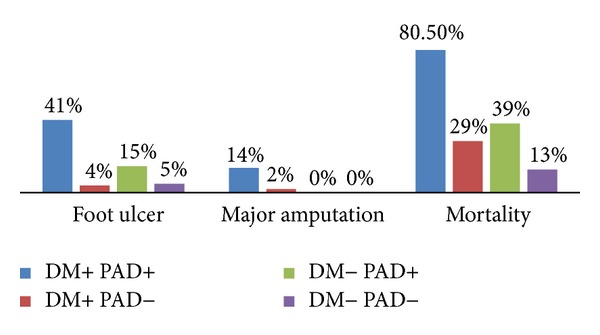
Outcomes in HD patients based on the presence/absence of DM and/or PAD (*P* = 0.001 for all).

In DM group, more number of patients developed PAD (61%).  Figure 3 demonstrates the outcomes in HD patients based on the presence/absence of DM and/or PAD.

Patients who had PAD showed increased incidence of FU (41% versus 4%; *P* = 0.001), major amputation (14% versus 2%; *P* = 0.02), and mortality (80.5% versus 29%; *P* = 0.001) than non-PAD. On the other hand, among non-DM patients only 29% developed PAD and the remaining 71% had no PAD. Of these patients, mortality rate was significantly higher among PAD group (39% versus 13%; *P* = 0.007) in comparison to non-PAD.

### 3.3. Univariate and Multivariate Logistic Regression Analysis

Univariate analysis (Figure 1) shows the mortality rate in patients with FU in both diabetic and nondiabetic patients.

**Figure 4:**
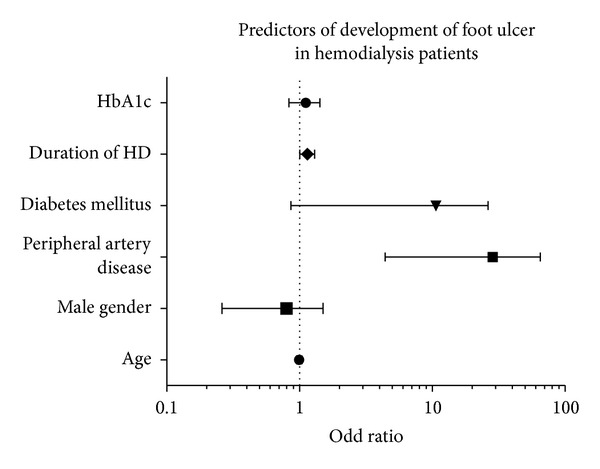


**Figure 5:**
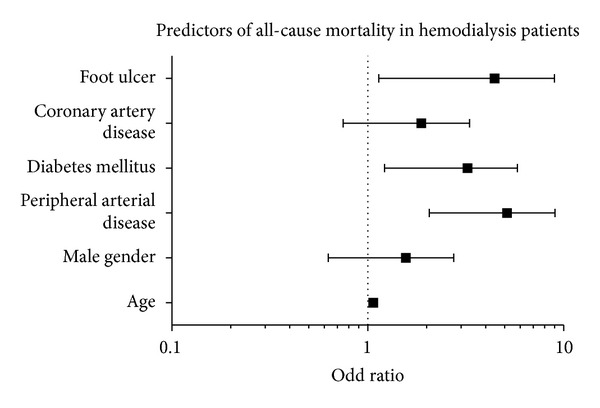


On multivariate analysis,after adjusting for DM, HbA1c, age, and gender, the presence of PAD was the major independent predictor of development of FU in HD patients with an adjusted odd ratio (aOR) of 16.0 (95% confidence interval (CI): 4.41–62.18, *P* = 0.001) followed by duration of HD with aOR 1.14 (95% CI: 1.005–1.299, *P* = 0.042). Furthermore, after adjusting for age, sex, and CAD, predictors of mortality in HD patients were PAD (aOR 4.1; 95% CI: 1.94–8.59, *P* = 0.001), FU (aOR 3.6; 95% CI: 1.28–10.002, *P* = 0.01), and DM (aOR 2.7, 95% CI: 1.23–5.89, *P* = 0.01) (Figures 4 and 5).

## 4. Discussion

The present study highlights the frequency and implications of FU in patients undergoing maintenance HD over a 5-year duration. There are several key findings in this report. In the entire HD cohort, 17% had FU. Also, among those who had FU, 17% had no DM. FU was diagnosed in 23% of diabetic HD patients. The mortality rates were higher in patients with FU in both diabetic and nondiabetic patients; however it was relatively higher in diabetic patients. Moreover, FU was associated with 4-fold increased risk of mortality after adjusting for age, sex, and CAD. Presence of PAD was associated with 16-fold increased risk of FU in HD after adjustment for age, sex, DM, and duration of HD. Patients in the FU group underwent higher number of repeated HD vascular accesses in comparison to non-FU group.

Recent studies have identified an increased risk of FU and LEA in CKD patients who did not receive renal replacement therapy [10, 17]. Other studies investigated patients of combined DM with ESRD and found a higher risk of FU in patients undergoing HD [11, 15]. A Swedish study demonstrated a 2.45 times increased risk of LEA in ESRD patients compared to those without ESRD [18]. Similarly, Prompers et al. [19] found the risk of nonhealing of FU to be 2.3-fold higher in ESRD than in non-ESRD patients.

The correlation between dialysis and foot complications among patients with DM and CKD has been initially described by McGrath and Curran [20]. They observed 50% mortality rate at one-year follow-up after LEA. In our study, the rate of major amputation was significantly higher in FU patients which corroborates with an earlier study showing increased rate of amputation in CKD patients undergoing dialysis (57%) as compared to those without dialysis (25%) [4]. The relevance of selecting FU in our report is that it is potentially preventable, and its progression generally leads to serious foot complications, major amputation, and mortality. In their long-term follow-up study (10 years), Morbach and coworkers [21] concluded that patients with diabetic foot had high mortality particularly in the presence of PAD or renal failure. In comparison to that study, our 5-year study showed that FU patients had higher mortality (81% versus 70.4%) although our patients were 6 years younger and less likely to have DM (83% versus 100%) and PAD (34% versus 55.5%). Moreover, the entire cohort of the present study was undergoing HD (100% versus 4%).

The association of severe complications in HD patients might be attributed to the cardiovascular risk factors. Several contributing factors have been proposed for the development of FU in patients with ESRD and DM. The important risk factors for the development of diabetic FU involve distal polyneuropathy, microangiopathy, and macroangiopathy. Also, cardiovascular autonomic neuropathy is another coexisting complication of DM [12]. O'Hare et al. [22] reported high incidence of PAD in HD patients which ranges from 24% to 77%. The authors found that PAD is independently associated with ESRD. According to one of our recent studies, PAD patients had 4- to 5-fold increased incidence of FU and LEA in comparison to non-PAD patients [14]. In our study, a higher incidence of retinopathy, polyvascular disease, angina, PAD, and nephropathy was associated with FU.

Ndip et al. [15] studied the risk factors associated with prevalent FU in patients with DM and CKD (predialysis versus on-dialysis). The authors reported that dialysis therapy and previous FU were the only independent predictors of the development of new FU. Kaminski et al. [23] reported a high prevalence of risk factors for FU present in patients with ESRD either with or without the coexistence of DM. The authors did not indicate the severity of ESRF or whether the patients were on dialysis or not.

Speckman et al. [24] found that DM, preexisting comorbidities, CVD, HD inadequacy, and lower serum albumin level are the major factors for LEA. Consistent with our findings, a recent meta-analysis reported higher association of CVD, DM, and FU [12]. Ischemic in comparison to neuropathic ulcers are associated with higher mortality rate. Moreover, the marked inflammatory response during the process of ulceration has a significant role in the initiation and worsening of the atherosclerosis [12]. In our study, among HD patients without diabetes, only 7% developed FU, despite a high prevalence of PAD and CAD.

Wolf et al. [17] reported that the presence of DM in ESRF patients increases the risk of LEA 10 times in comparison to those who are diabetic without ESRF. Moreover, during HD, around 4% of patients require an amputation each year [17, 25].

Our subanalysis showed significantly higher incidence of FU, amputation, and mortality in diabetic patients than in non-DM patients. Also, patients with combined DM and PAD revealed increased association of FU, amputation, and mortality. Our findings are supported by a large meta-analysis which showed that PAD is independently associated with CVD and all-cause mortality [12]. Further, the authors reported an increased risk of all-cause mortality in diabetic patients who developed FU than in diabetics without FU.

This study has several limitations. Due to retrospective nature of the study, it is not possible to specify the extent of infection, neuropathy, ischemia, depth, or extent of tissue loss grade of FU. Another limitation is the additive effect of diabetes on HD patients who developed foot ulcers. We did not know how many HD patients developed DM during the follow-up. In order to confirm our findings, large sample-sized studies are needed to establish the implications of FU in HD patients. ABI < 9 was used as a part of the diagnosis of PAD which may lead to underestimation of the disease. Previous data showed that as with low ABI, high rates of mortality, vascular events, and amputation were reported in patients with high ABI or noncompressible vessels. In a large study of patients with DM and CAD, Singh et al. [26] reported a high prevalence of arterial stiffness, similar to that seen in older individuals and dialysis patients. Recently, Yap et al. (2014) defined PAD as an ABI < 0.9 or >1.4, these high ABI values were observed in patients with diabetes, particularly for those with concomitant CKD [27].

In conclusion, hemodialysis is a significant risk factor for FU which needs special attention. Further, PAD is significantly associated with FU, amputation, and mortality in diabetic HD patients. The increased risk of mortality could be explained by the greater burden of CVD in these patients. Therefore, HD patient needs intensive foot care to avoid complications of the lower limb and warrant progressive modification of CVD risk factors.

## Figures and Tables

**Table 1:** Foot ulcer in hemodialysis patients (*n* = 252).

	No-FU (*n* = 210)	FU (*n* = 42)	*P* value
Age	56 ± 16	62 ± 9	0.02
Males (%)	49	55	0.49
BMI	27.2 ± 7.1	28.6 ± 9.2	0.35
Married (%)	85	95	0.07
Illiterate (%)	43	50	0.39
Duration of Hemodialysis (yrs)	6.4 ± 5.2	6.2 ± 4.7	0.84
Prior Renal transplant	14%	2.4%	0.03
Dyslipidemia (%)	24	33	0.19
Hypertension (%)	84	83	0.93
Smoking (%)	4.3	7.1	0.43
Nephropathy (%)	42	79	0.001
Diabetes Mellitus	56%	83.3%	0.001
HbA1c	6.9 ± 1.7	7.2 ± 1.8	0.31
Retinopathy	54%	88%	0.001
≥3 vascular accesses	47%	69%	0.01
AV Fistula	72%	69%	0.71
Tunnel Catheter	82%	17%	0.36
Major amputation	2%	36%	0.001
Coronary artery disease	45.7%	81%	0.001
Peripheral artery disease	38.5%	87.8%	0.001
Total Renal transplant	11%	2%	0.001
Total 5-year deaths	43%	81%	0.001

FU: Foot ulcer; CAD: Coronary artery disease.

**Table 2:** Laboratory results.

	No ulcer	Ulcer	*P* value
Cholesterol (mean ± SD)	4.2 ± 1.1	4.1 ± 0.8	0.66
Triglyceride (mean ± SD)	1.9 ± 1.4	1.9 ± 1.1	0.99
HbA1c (mean ± SD)	6.9 ± 1.7	7.2 ± 1.8	0.32
Hemoglobin (mean ± SD)	11.3 ± 1.9	11.5 ± 1.6	0.46
Vitamin D (mean ± SD)	13.2 ± 10	13.3 ± 6	0.98
Serum calcium (mean ± SD)	2.09 ± 0.23	2.13 ± 0.21	0.22
Phosphorus (mean ± SD)	1.55 ± 0.53	1.62 ± 0.45	0.37
Albumin (mean ± SD)	37 ± 5	35 ± 5	0.15
PTH (mean ± SD)	423 ± 404	526 ± 460	0.18
